# Laboratory markers of severity across three COVID-19 outbreaks in Australia: has Omicron and vaccinations changed disease presentation?

**DOI:** 10.1007/s11739-022-03081-y

**Published:** 2022-09-14

**Authors:** Julie Wang, Kay Weng Choy, Hui Yin Lim, Prahlad Ho

**Affiliations:** 1 Northern Health, Northern Hospital, 185 Cooper St Epping, Melbourne, VIC 3076 Australia; 2 Northern Pathology Victoria, Epping, VIC Australia; 3 University of Melbourne, Parkville, VIC Australia

**Keywords:** COVID-19, Omicron, Laboratory biomarkers, Risk prediction model

## Abstract

COVID-19 has rapidly evolved since it was first discovered in December 2019. We aimed to retrospectively review our experience with COVID-19 infection across 2020–2022, focusing on differences in laboratory markers at presentation. Consecutive adult patients admitted to hospital with confirmed COVID-19 infection were retrospectively reviewed across three periods (29/3/2020–29/9/2020, 16/8/2021–13/10/2021 and 1/1/2022–31/1/2022), correlating with the lineages B.1.338, Delta (B.1.617.2) and Omicron (B.1.1.159), respectively. Laboratory findings of the first requested blood test within 24 h of presentation were recorded and correlated with patient outcome. The primary outcome was requirement for oxygen therapy at any point. Inflammatory markers, namely serum ferritin, lactate dehydrogenase (LDH), C-reactive protein (CRP) were significantly lower on presentation during 2022 compared to 2021, corresponding to a milder disease course. More than 80% of 2022 patients had received 2 or more vaccine doses and fully vaccinated patients displayed significantly lower inflammatory markers at presentation. Using 2022 data, a multivariate prediction model was constructed to predict for oxygen requirement, with *c*-statistic 0.86. Patients in 2022, corresponding with the Omicron variant, displayed a milder disease course, even in hospitalised patients, with the majority not requiring oxygen and lower inflammatory markers. We constructed a simple-to-use risk prediction model with *c*-statistic 0.86 which may identify individuals who can be safely managed as outpatients in the era of highly transmissible variants.

## Background

The severe acute respiratory syndrome-associated coronavirus 2 (SARS-CoV-2) has evolved since its emergence in Wuhan China in December 2019. The presentation of COVID-19 has changed as a result of the emergence of different variants [1, 2], and the advent of vaccines which dramatically reduce the severity of disease. The Omicron variant (B.1.1.529), first reported as a variant of concern on 26 November 2021 [2] due to its many mutations, has now become the dominant lineage globally [3]. Fortunately, the Omicron waves globally have been markedly different and characterised by increased transmissibility, but a milder disease course compared to the previous Delta variant [4, 5]. Nonetheless, Omicron remains a potentially severe disease in the unvaccinated, elderly [6] and immunocompromised who have reduced vaccine antibody response, and impaired cellular mediated immunity [7].

The reduction in severe disease may also be in part due to widespread vaccinations in some countries resulting in a ‘decoupling’ of infections with reduced hospitalisations and mortality [8]. Australia has now a high vaccine coverage, and Melbourne attained 90% vaccine coverage in those over 12 years old on 30 November 2021 [9]. Nevertheless, COVID-19 remains a disruptive infection and one that is intensive in its utilisation of healthcare resources, particularly with the resurgence of other viruses such as influenza. Identifying patients more likely to have a mild disease course and, therefore, not needing oxygenation support may be a way to rationalise healthcare resources as these patients could be treated in the outpatient setting and potentially not require hospitalisation.

COVID-19 has spread differently in Australia than in many other countries [10]. Australia’s unique geographical location as a large island continent in the southern hemisphere allowed the strict implementation of border controls that limited the importation of SARS-Cov-2 and enabled the pursuit of a ‘zero-COVID’ strategy through local lockdowns and contact tracing. Due to these distinctive characteristics, Melbourne, where this study is conducted, saw very few COVID-19 infections up until May 2020, when a breach of border control led to the first major outbreak that was ultimately contained with lockdown measures until its lift in October 2020. There was again no local transmission of the virus until mid-2021, when the more contagious Delta strain produced the second significant outbreak. The ‘zero-COVID’ approach was then abandoned in December 2021 once the population had attained high vaccination coverage. Shortly following this, the Omicron variant was detected in Melbourne in December 2021, and has been responsible for a large increase in infections that persists to the present day.

In this study, we review our experiences with three distinct COVID outbreaks in patients presenting to the Northern Hospital, a major tertiary hospital in Melbourne, specifically focusing on differences in laboratory markers at presentation and correlating these with outcomes. Using the data from 2022, we set out to develop a new pilot risk model to risk stratify for oxygen requirement within a highly vaccinated cohort in the Omicron era.

## Methods

Data were collected retrospectively across 3 periods of the COVID-19 pandemic at the Northern Hospital, a sub-tertiary level hospital in the northern metropolitan area of Melbourne, Australia. The collection periods comprised 29 March 2020–29 September 2020, 16 August 2021–13 October 2021 and 1–31 January 2022. These correlated with outbreaks of the B.1.338 [11], Delta (B.1.617.2) [12], and Omicron (B.1.1.159) [13] virus subtypes, respectively. Consecutive adult patients (aged ≥18 years old) with confirmed SARS-CoV-2 on polymerase chain reaction (PCR) test result, within 5 days of hospital admission, were identified via the hospital electronic medical records system. Laboratory parameters of the first requested blood test within 24 h of presentation to the hospital were recorded. Medical records were retrospectively reviewed, and data collected included patient demographics, co-morbidities, vaccination status for SARS-CoV-2, treatments (including requirement for oxygen at any point during the admission) and patient outcomes. Patients were followed up from time of hospital admission to discharge or death, with follow-up censored at time of hospital discharge.

The primary outcome was requirement for oxygen therapy at any point during the hospital admission. Secondary outcome was in-hospital clinical deterioration, defined as the requirement of ventilatory support (high-flow oxygen, non-invasive and invasive ventilation), admission to intensive care unit, or death attributed to COVID-19 infection. Asymptomatic patients were defined as those patients without symptoms of COVID-19 throughout their hospital admission and were admitted for another indication. The patients were considered fully vaccinated if they had received two or more doses of vaccinations for SARS-CoV-2. Routine booster vaccinations were only routinely available from December 2021 onwards. Venous thromboembolism (VTE) was defined as an episode of deep vein thrombosis (DVT), or pulmonary embolus (PE) objectively diagnosed during the admission episode. Arterial thrombotic event was defined as an acute myocardial infarction, cerebrovascular accident, or another objectively diagnosed episode of thrombotic event within the arterial vasculature. Death was defined as being caused by COVID-19 if this was listed as the primary cause of death in the death certificate.

The Northern Health Office of Research approved the study as a quality improvement audit (ALR 69.2020).

### Statistical analysis

Statistical analysis was performed using Stata version 17.0 (StataCorp, College Stations, Texas, USA). Comparisons between patient groups were conducted using Student’s *t*-tests for normally distributed variables and presented as means and standard deviation. Mann–Whitney (rank-sum) was performed for non-normally distributed variables and presented as medians and interquartile ranges (IQR). Categorical variables were presented as counts and frequencies with chi-squared tests or ANOVA to test for differences.

Data from the 2022 collection period were used to derive a multivariate predictive model, with oxygen requirement as the primary endpoint. Univariate logistic regression was first conducted to identify variables associated with oxygen requirement. Variables with *p*-values less than 0.2 were considered in the multivariable analysis. Missing values were addressed by multiple imputation with chained equations. Multicollinearity was assessed between variables and urea was determined to be a highly multicollinear variable and not included in subsequent multivariate analyses. Backwards stepwise logistic regression was used to identify candidate final multivariate models. *C*-statistics (area under the receiver operating curve), Schwarz’ Bayesian Information Criterion, Akaike Information Criterion and the Hosmer–Lemeshow test were used to compare model fit and select the preferred model. Statistical significance was set at a *p*-value of less than 0.05.

## Results

### Baseline demographics

A total of 196, 419 and 467 patients were hospitalised with COVID-19 infection during the 2020, 2021 and 2022 collection periods, respectively. During these periods, the number of SARS-CoV-2-infected persons notified to the Victorian Department of Health from our local catchment areas (Moreland, Whittlesea, Hume) were 3756 in 2020, 14,805 in 2021 and 49,447 in 2022 [14]. Using these data, the rates of hospitalisation for 2020, 2021 and 2022 were approximately 5.22%, 2.83% and 0.94%, respectively.

Table 1 shows attributes of hospitalised COVID-19 patients over the 3 collection periods. Compared to 2021, patients in the 2022 cohort were significantly older (median 70 years vs 55 years, *p* < 0.001) but more likely to be fully vaccinated (80.2% vs. 6.4%, *p* < 0.001). Rates of clinical deterioration and death were the lowest in 2022 (22.9% and 5.9%, respectively). After adjustment for age, inflammatory markers, namely serum ferritin, lactate dehydrogenase (LDH), C-reactive protein (CRP) and plasma fibrinogen were significantly lower on presentation during 2022 compared to 2021. There were no significant differences for D-dimer between 2021 and 2022. Compared to other years, a high number of patients in 2020 were from residential care facilities (31.6% in 2020, 1.0% in 2020 and 6.6% in 2022, *p* < 0.001).

**Table 1:** Baseline characteristics, laboratory results and clinical outcomes of 1082 patients categorised by the respective COVID-19 waves of infection

	2020	2021	2022	*p*-value		
				All*	2020 vs 2022	2021 vs 2022*
*N* (% of local infections)	196 (5.2%)	419 (2.8%)	467 (0.9%)			
Number of infected persons notified in local catchment area	3756	14,805	49,447			
Age (years)	69.5 (48.0, 84.0)	55.0 (40.0, 71.0)	70.0 (47.0, 82.0)	** <0.001**	0.46	** <0.001**
Male	95 (48.5%)	203 (48.4%)	220 (48.0%)	0.74	0.92	0.66
Residential care	62 (31.6%)	4 (1.0%)	31 (6.6%)	** <0.001**	** <0.001**	** <0.01**
Fully vaccinated (≥2 doses)	0 (0.0%)	25 (6.4%)	365 (80.2%)	** <0.001**	** <0.001**	** <0.001**
Days from symptom onset to admission	N/A^a^	6.0(3.0, 8.0)	4(2.0, 8.0)	N/A^a^	N/A^a^	**0.01**
Co-morbidities						
Hypertension	110 (56.1%)	163 (38.9%)	219 (47.0%)	**0.020**	**0.032**	0.44
Diabetes	61 (31.1%)	113 (27.0%)	166 (35.6%)	0.40	0.27	0.59
COPD	11 (5.6%)	24 (5.7%)	37 (7.9%)	0.51	0.29	0.50
IHD	31 (15.8%)	44 (10.5%)	89 (19.1%)	0.69	0.32	0.45
CKD	19 (9.7%)	22 (5.3%)	71 (15.2%)	** <0.018**	0.058	**0.02**
CCF	26 (13.3%)	22 (5.3%)	51 (10.9%)	0.22	0.40	0.46
Malignancy	3 (1.5%)	18 (4.3%)	38 (8.2%)	**0.003**	**0.001**	0.09
Smoking history				**0.006**	**0.001**	0.83
Non-smoker	157 (80.1%)	254 (66.5%)	265 (65.4%)			
Smoker	9 (4.6%)	58 (15.2%)	38 (9.4%)			
Ex-smoker	30 (15.3%)	70 (18.3%)	102 (25.2%)			
BMI (kg/m^2^)	27.2 (23.6, 32.0)	29.9 (26.9, 35.1)	28.4 (25.1, 33.9)	**0.003**	**0.016**	0.54
Outcomes						
Venous thromboembolism	4 (2.0%)	13 (3.1%)	10 (2.2%)	0.91	0.91	0.86
Arterial thrombus	0 (0.0%)	10 (2.4%)	21 (4.5%)	**0.010**	**0.003**	0.60
Assisted ventilation	25 (12.8%)	134 (32.0%)	90 (19.3%)	** <0.001**	**0.043**	** <0.001**
Intensive care unit	20 (10.2%)	61 (14.6%)	34 (7.3%)	**0.028**	0.21	**0.005**
Clinical deterioration	65 (33.2%)	160 (38.2%)	107 (22.9%)	** <0.001**	**0.006**	** <0.001**
Death caused by COVID-19	42 (21.4%)	46 (11.1%)	27 (5.9%)	** <0.001**	** <0.001**	** <0.001**
Laboratory findings						
Haemoglobin (g/L), mean (SD)	132.7 (19.6)	137.9 (18.6)	132.5 (20.0)	0.29	0.93	0.12
Neutrophils (×10^9^/L)	4.4 (3.0, 6.2)	4.1 (2.9, 5.9)	4.8 (3.4, 7.0)	**0.002**	0.09	** <0.001**
Lymphocytes (×10^9^/L)	0.9 (0.7, 1.5)	1.0 (0.7, 1.3)	1.0 (0.7, 1.6)	0.19	0.97	0.17
Platelets (×10^9^/L)	225 (176, 277)	208 (163, 262)	215 (162, 272)	0.22	0.38	0.13
Creatinine (mmol/L),	76.5 (59.0, 100.0)	72.0 (58.0, 90.0)	80.0 (62.0, 113.0)	**0.016**	**0.029**	**0.006**
Ferritin (ug/L)	349.0 (169.0, 919.0)	579.0 (241.0, 1197.0)	334.0 (132.0, 777.0)	** <0.001**	0.41	** <0.001**
LDH (units/L)	287.0 (213.0, 356.0)	361.0 (263.0, 496.0)	296.0 (221.0, 397.0)	** <0.001**	0.28	** <0.001**
CRP (mg/L)	49.5 (13.0, 96.5)	58.0 (23.0, 114.0)	33.0 (9.0, 92.0)	**0.001**	0.16	**0.002**
D-dimer (mg/L FEU)	0.7 (0.5, 1.2)	0.9 (0.6, 1.4)	0.9 (0.5, 1.8)	0.45	**0.007**	0.32
Fibrinogen (g/L)	5.7 (4.7, 6.8)	5.3 (4.5, 6.5)	4.7 (3.9, 6.4)	**0.003**	** <0.001**	**0.015**

*COPD* Chronic obstructive pulmonary disease, *IHD* Ischaemic heart disease, *CKD* Chronic kidney disease, *CCF* Congestive cardiac failure, *BMI* Body mass index, *LDH* Lactate dehydrogenase, *CRP* C-reactive protein

*p-values are adjusted for age; boldened values signify *p* < 0.05

^a^Date of symptom onset unavailable for 2020 cohort

Data are *n* (%), and median (interquartile range, IQR), unless specified otherwise

### Analysis of patients admitted during the 2022 collection period

**Fig. 1:**
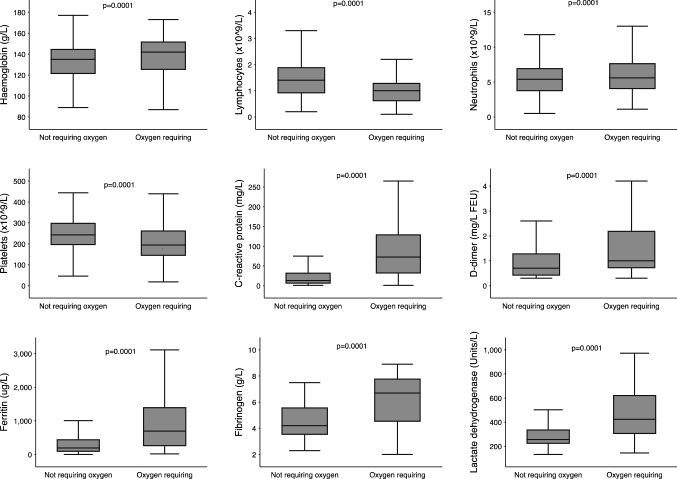
Boxplots of laboratory findings on admission according to oxygen requirement. *P*-values were derived from the Kruskal–Wallis method

Symptomatic patients were older compared to those who were asymptomatic (71.8 vs 41.0 years, *p* < 0.001). There were 256 patients (54.8%) who did not require oxygen therapy in 2022 and 105 patients (22.5%) who were asymptomatic. Table 2 displays patient characteristics and laboratory results according to outcomes. Laboratory markers on admission appear to be associated with likelihood of requiring oxygen (Fig. 1), as well as correlated with patient outcomes, with asymptomatic patients displaying the highest lymphocyte count and lowest ferritin, LDH and CRP at presentation.

**Table 2:** - Baseline characteristics, laboratory data and clinical outcomes of 467 patients categorised by disease severity in 2022

	Asymptomatic	Symptomatic but did not require oxygen therapy	Required oxygen therapy but did not deteriorate	Clinically deteriorated	*p*-value*
*N*	105	151	104	107	
Age (years)	41.0 (32.0, 74.0)	68.0 (40.0, 82.0)	73.5 (66.5, 80.0)	74.0 (61.0, 83.0)	** <0.001**
Male	44 (42.7%)	64 (43.2%)	50 (49.5%)	62 (58.5%)	**0.042**
Fully vaccinated (≥2 doses)	78 (83.0%)	127 (84.7%)	88 (84.6%)	72 (67.3%)	**0.001**
Vaccine type					** <0.041**
Astra Zeneca (Vaxzevria)	25 (32.1%)	53 (43.1%)	52 (65.0%)	48 (64.9%)	
mRNA (Pfizer-BioNTech or Moderna)	53 (67.9%)	70 (56.9%)	28 (35.0%)	26 (35.1%)	
Days from admission to last vaccine	90.0 (69.0, 104.0)	93.0 (71.0, 125.0)	92.0 (63.0, 130.0)	98.0 (73.0, 145.0)	0.06
Days from symptom onset to admission	N/A	3.0 (2.0, 6.0)	6.0 (3.0, 9.0)	5.0 (2.5, 8.0)	0.053
COPD	2 (1.9%)	6 (4.0%)	14 (13.5%)	15 (14.2%)	**0.007**
Smoking history					** <0.001**
Non-smoker	71 (75.5%)	95 (74.8%)	52 (56.5%)	47 (51.1%)	
Smoker	11 (11.7%)	10 (7.9%)	10 (10.9%)	7 (7.6%)	
Ex-smoker	12 (12.8%)	22 (17.3%)	30 (32.6%)	38 (41.3%)	
BMI (kg/m^2^)	26.3 (24.4, 30.4)	28.3 (24.7, 33.3)	29.1 (25.2, 36.5)	29.4 (26.3, 34.3)	**0.005**
Outcomes					
Venous thromboembolism	4 (3.9%)	4 (2.7%)	0 (0.0%)	2 (1.9%)	0.50
Arterial thrombus	2 (1.9%)	3 (2.0%)	12 (11.5%)	4 (3.7%)	0.48
Assisted ventilation	0 (0.0%)	0 (0.0%)	0 (0.0%)	90 (84.1%)	** <0.001**
Intensive care unit	0 (0.0%)	0 (0.0%)	0 (0.0%)	34 (31.8%)	** <0.001**
Clinical deterioration	0 (0.0%)	0 (0.0%)	0 (0.0%)	107 (100.0%)	** <0.001**
Death caused by COVID-19	0 (0.0%)	0 (0.0%)	0 (0.0%)	27 (28.1%)	** <0.001**
Laboratory findings					
Haemoglobin (g/L), mean (SD)	133.3 (22.2)	129.0 (18.8)	137.0 (18.2)	132.4 (20.2)	**0.026**
Neutrophils (×10^9^/L)	5.5 (3.9, 7.4)	4.3 (3.1, 6.3)	4.9 (3.5, 6.8)	5.0 (3.4, 7.6)	0.43
Lymphocytes (×10^9^/L)	1.5 (1.0, 2.1)	1.0 (0.6, 1.6)	0.9 (0.6, 1.2)	0.9 (0.6, 1.3)	** <0.001**
Platelets (×10^9^/L)	249.5 (202.0, 300.0)	223.0 (168.0, 286.0)	195.0 (150.0, 233.0)	193.0 (147.0, 256.0)	**0.001**
Neutrophil/Lymphocyte ratio	4.6 (2.4, 8.3)	4.2 (2.8, 7.8)	4.8 (3.2, 9.0)	4.6 (2.9, 8.0)	0.10
Creatinine (mmol/L)	68.5 (58.5, 91.0)	80.0 (59.5, 114.0)	87.0 (66.5, 115.5)	92.0 (68.0, 141.0)	0.09
Ferritin (ug/L)	152.0 (66.0, 281.0)	212.0 (82.0, 451.0)	491.0 (264.0, 907.0)	576.0 (267.0, 1238.5)	** <0.001**
LDH (units/L)	228.0 (187.0, 296.0)	253.0 (209.0, 330.0)	312.0 (244.0, 397.0)	398.0 (284.0, 503.0)	** <0.001**
CRP (mg/L)	9.0 (3.0, 26.0)	18.0 (7.0, 48.0)	65.0 (23.0, 120.5)	73.0 (33.0, 130.0)	** <0.001**
D-dimer (mg/L FEU)	0.8 (0.4, 1.5)	0.8 (0.4, 1.4)	0.9 (0.6, 1.8)	1.2 (0.6, 2.9)	0.15
Fibrinogen (g/L)	5.0 (4.0, 5.8)	4.1 (3.4, 5.0)	5.1 (4.4, 7.0)	5.3 (4.0, 6.7)	0.10

*mRNA* Messenger RNA, *COPD* Chronic obstructive pulmonary disease, *IHD* Ischaemic heart disease, *CKD* Chronic kidney disease, *CCF* Congestive cardiac failure, *BMI* Body mass index, *LDH* Lactate dehydrogenase, *CRP* C-reactive protein

^*^All *p*-values are adjusted for age; boldened values signify *p* < 0.05

Data are *n* (%), and median (interquartile range, IQR), unless specified otherwise

Table 3 displays patient characteristics, laboratory results and clinical outcomes according to vaccination status in 2022. Patients who received mRNA vaccines (Pfizer-BioNTech (Comirnaty) or Moderna (Spikevax)) comprised 49.4% of fully vaccinated individuals. Fully vaccinated patients were significantly less likely to experience clinical deterioration (19.7% vs 38.9%, *p* < 0.001) or die (3.9% vs 15.1%, *p* < 0.001). Fully vaccinated patients also displayed significantly lower ferritin, LDH, CRP and D-dimer at presentation.

**Table 3:** Baseline characteristics, laboratory results and clinical outcomes of 455 patients categorised by vaccination status in 2022

	0 or 1 dose	2 or 3 doses	*p*-value*
*N*	90	365	
Age (years)	68.5 (41.0, 82.0)	71.0 (50.0, 82.0)	0.47
Male	41 (46.6%)	172 (48.0%)	0.81
Vaccination status			** <0.001**
Unvaccinated	77 (85.6%)	0 (0.0%)	
1 dose	13 (14.4%)	0 (0.0%)	
2 doses	0 (0.0%)	333 (91.2%)	
3 doses	0 (0.0%)	32 (8.8%)	
Vaccine type			0.39
Astra Zeneca (Vaxzevria)	5 (38.5%)	173 (50.6%)	
mRNA (Pfizer-BioNTech or Moderna)	8 (61.5%)	169 (49.4%)	
Days from symptom onset to admission	4.0 (2.0, 8.0)	4.0 (2.0, 8.0)	0.91
Diabetes	23 (25.8%)	140 (38.4%)	**0.027**
BMI (kg/m^2^)	30.3 (25.6, 37.3)	28.2 (25.1, 33.8)	0.22
Outcomes			
Venous thrombosis	2 (2.3%)	7 (2.0%)	0.85
Arterial thrombosis	3 (3.3%)	18 (4.9%)	0.52
Assisted ventilation	30 (33.3%)	60 (16.4%)	** <0.001**
Clinical deterioration	35 (38.9%)	72 (19.7%)	** <0.001**
Death caused by COVID-19	13 (15.1%)	14 (3.9%)	** <0.001**
Laboratory findings			
Haemoglobin (g/L), mean (SD)	133.6 (17.6)	132.4 (20.3)	0.61
Neutrophils (×10^9^/L)	4.5 (3.3, 7.6)	4.8 (3.4, 6.8)	0.91
Lymphocytes (×10^9^/L)	0.9 (0.6, 1.4)	1.0 (0.7, 1.6)	0.091
Platelets (×10^9^/L)			
Creatinine (mmol/L)	76.0 (58.0, 103.0)	84.0 (63.0, 116.0)	0.097
Ferritin (ug/L)	631.0 (264.0, 1406.0)	306.0 (123.0, 621.0)	** <0.001**
LDH (units/L)	327.0 (246.0, 486.0)	283.0 (219.5, 375.5)	**0.004**
CRP (mg/L)	62.0 (18.0, 104.0)	30.0 (9.0, 88.0)	**0.011**
D-dimer (mg/L FEU)	1.2 (0.7, 2.1)	0.8 (0.5, 1.8)	**0.011**
Fibrinogen (g/L)	5.1 (4.5, 6.5)	4.6 (3.9, 6.2)	0.33

*mRNA* Messenger RNA, *BMI* Body mass index, *LDH* Lactate dehydrogenase, *CRP* C-reactive protein

^*^All *p*-values are univariate; boldened values signify *p* < 0.05

Data are *n* (%), and median (interquartile range, IQR), unless specified otherwise

### Prediction model for oxygen requirement using 2022 data

**Fig. 2:**
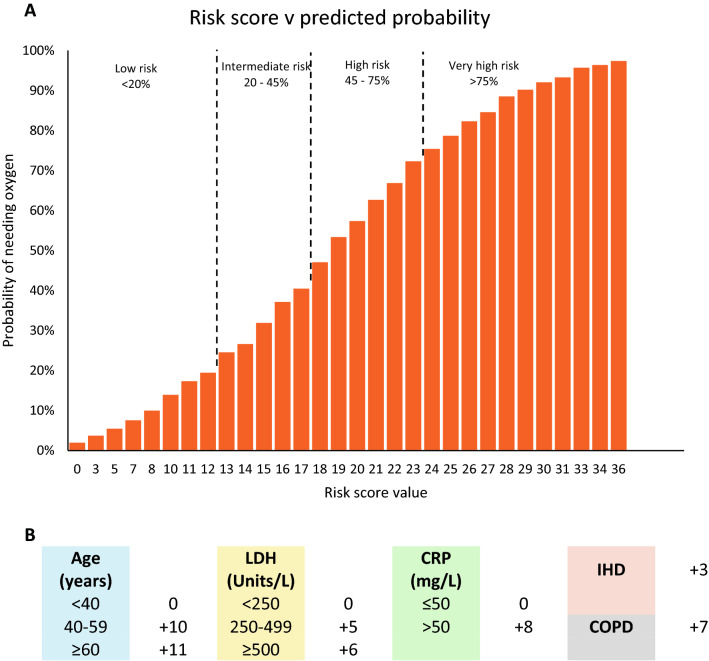
**a** Predicted probability of oxygen requirement against risk score **b** Simple scoring system for predictive model

Using available data from the 2022 collection period, a multivariate prediction model for oxygen requirement was constructed using logistic regression. The final multivariate prediction model for oxygen requirement is displayed in Table 4. The model was converted to a simple scoring system for bedside use (Fig. 2b), with suggested risk categorisation based on predicted probability (Fig. 2a).

**Table 4:** Multivariate logistic model for prediction of oxygen requirement

	Odds Ratio	Co-efficients	Standard error of odds ratio	*t*-score	*p* > *t*	95% confidence interval of odds ratio	
Age (years)							
<40	1						
40–59	8.58	2.15	4.84	3.81	** <0.001**	2.84	25.89
≥60	10.24	2.33	5.11	4.66	** <0.001**	3.85	27.24
LDH (Units/L)							
<250	1.00						
250–499	2.87	1.06	0.84	3.61	** <0.001**	1.62	5.10
≥500	3.96	1.38	2.01	2.71	**0.007**	1.46	10.77
CRP (mg/L)							
≤50	1.00						
>50	5.57	1.72	1.51	6.32	** <0.001**	3.27	9.48
IHD	1.91	0.65	0.59	2.12	**0.034**	1.05	3.49
COPD	4.19	1.44	2.06	2.92	**0.003**	1.60	10.98

*COPD* Chronic obstructive pulmonary disease; *IHD* Ischaemic heart disease, *LDH* Lactate dehydrogenase, *CRP* C-reactive protein

## Discussion

In this retrospective study from Melbourne, Australia, we have demonstrated the 2022 COVID-19 outbreak, caused largely by the Omicron variant, is associated with a significantly milder clinical presentation and a lower hospitalisation rate, as well as markedly lower inflammatory markers within 24 h of hospital presentation. We observed that abnormal inflammatory markers, particularly CRP, ferritin and LDH to be associated with a greater risk of requiring oxygen therapy even in 2022, where most hospitalised patients had Omicron. Interestingly, patients who were not vaccinated in 2022 showed inflammatory markers which were significantly higher than the vaccinated and were similar to what was seen in the unvaccinated population in previous collection periods.

Growing epidemiological data supports the Omicron variant being much milder compared to previous waves [4, 15, 16]. Possible reasons for the milder disease include evidence that Omicron replicates less efficiently in the lung parenchyma [17], and persistence of effective T-cell-mediated immunity from previous infection or vaccinations, as the T-cell epitopes in Omicron remain substantially conserved [18]. The experience with COVID-19 infections in our local catchment area in 2022 has been a lower proportion of patients requiring admission and improved patient outcomes compared to earlier years. This is likely due to the combination of the milder Omicron variant and widespread vaccination as well as improved knowledge and experience in the management of COVID-19. Improved treatments for hospitalised patients may also have played a role, with antiviral remdesivir, JAK inhibitor baricitinib, and monoclonal antibodies like sotrovimab being widely used by the time of the 2022 study period. Despite the highest recorded number of reported infections during the 2022 study period, hospital admissions in our centre accounted for only about 0.94% of total infections reported from the local catchment area, compared to 2.83% in 2021 (Table 1). The highest hospitalisation rate (5.2%) was observed during the 2020 collection period, which was due to significant transmission among residents of aged care facilities at the time. Residents of aged care facilities comprised 31.6% of patients in the 2020 study period (Table 1), which also explains the high death rate and the relatively low number of people who received assisted ventilation compared to other study periods.

The availability of vaccinations was likely a major contributor to the improved outcomes observed in 2022. In our study, the vaccination rate increased from 6.4% in the 2021 period to 80.2% fully vaccinated (defined as receiving at least 2 doses) in the 2022 study period. In keeping with trends in published literature, vaccinated patients in our 2022 cohort were observed to be significantly less likely to require assisted ventilation (16.4% vs 33.3%, *p* < 0.001) or die (3.9% vs 15.1%, *p* < 0.001). Interestingly, we observed that inflammatory markers in fully vaccinated patients, including ferritin, LDH, CRP and D-dimer to be significantly lower in fully vaccinated patients even at presentation (Table 3). Conversely, the unvaccinated patients showed inflammatory marker patterns like previous waves and may suggest that vaccination directly impacts the inflammatory response to SARS-CoV-2.

Previous research has identified lymphopenia, thrombocytopenia, increased D-dimer, LDH, and CRP as indicators and predictors of severe COVID-19 infection [19, 20]. Very few studies have examined differences in laboratory markers in vaccinated or Omicron-infected cohorts. A multicentre study of 1716 patients [21] presenting to emergency departments in Paris, France found CRP at presentation in Omicron-infected patients to be significantly lower than those with the Delta variant (21.0 vs 61.9 mg/L, difference 40.9 (CI 32.2–49.6)). We found similar findings in the 2022 wave with lower median CRP (33.0 vs 58.0, *p* = 0.002), ferritin (334.0 vs 579.0, *p* < 0.001) and LDH (296.0 vs 361.0, *p* < 0.001) compared to the 2021 wave, and correlates with the mild clinical phenotype associated with Omicron. Interestingly, despite this mild clinical presentation, the presence of abnormal inflammatory laboratory markers strongly correlated with requiring oxygen (Table 4).

Given the changed clinical phenotype with Omicron and the majority (54%) of 2022 patients did not require oxygen at any point in their admission, we created a new predictive risk model using data from 2022, focusing on patients at risk of developing disease severe enough to require hospital care, defined broadly as the need for oxygen. Patients deemed at low risk could potentially be managed in the outpatient setting or in subacute facilities, thereby reducing demands on healthcare resources. The resulting multivariate model had a *c*-statistic of 0.86, and key parameters were age, CRP, LDH, and presence of ischaemic heart disease or chronic obstructive pulmonary disease. Relatively few models have focused on oxygen requirement as the target outcome of COVID-19 infection. Lee and colleagues [22] developed the “CHANeL” prediction model based on serial CRP, neutrophil, and lymphocyte counts during the first 3 days of hospitalisation, along with age and hypertension status, which provide a reliable estimate of the risk of supplement oxygen requirement among patients hospitalised with COVID-19. However, we did not find neutrophil and lymphocyte counts to be particularly discerning for risks of clinical deterioration. Our model has the advantage of being easy to use with a simple scoring system and with few variables using readily available laboratory tests at a single timepoint at presentation. It also does not require any knowledge of radiological findings, or clinical findings such as respiratory rate which may be dynamic and rapidly changing even at presentation.

We acknowledge the potential limitations associated with the retrospective nature of this study. This study may be subject to treatment selection biases, quality of medical records and incomplete datasets due to lack of documentation. We also acknowledge the relatively small sample sizes compared to larger international studies and the unique situation in Australia where there were relatively well-controlled COVID outbreaks in 2020 and 2021 with limited numbers and deaths. Despite the absence of accurate local variant data, it is noted that the Omicron variant was first detected in Victoria, Australia in mid-December 2021, and by January 2022 comprised at least 80% of positive COVID samples [13] and accordingly, we extrapolate that most of the patients in the 2022 study period were infected with the Omicron variant. We are also mindful of recent criticisms directed at risk prediction models for COVID-19 [23] and that ideally, any risk model should be prospectively validated and evaluated in external settings to account for differences in healthcare systems. Prospective validation of this risk model is currently underway in our institution. Our results are also reported in the hope that it will provide a simple case-based model on which future collaborative efforts can build upon. Efforts to aggregate laboratory data across international health systems will provide an opportunity to explore such transformations if individual-level data become accessible.

## Conclusion

In this analysis of three distinct outbreaks of the COVID-19 outbreak in Melbourne, Australia, we found significant differences in patient characteristics, laboratory results on presentation and clinical outcomes across 2020–2022. In a highly vaccinated and Omicron-dominant inpatient setting, patients in the most recent 2022 outbreak displayed a relatively milder disease course, with more than 54% not requiring oxygen therapy. We propose a simple-to-use risk prediction model derived from the 2022 data, a sample with high rates of vaccination and with Omicron as the dominant variant, with the focus on identifying patients who may be safely treated at home. Prospective validation of this risk model is currently underway at our institution.

## Declarations

### Human and animal right statement

Please provide ethical statement for human and animal right statement.
